# Targeting non-coding RNAs to overcome cancer therapy resistance

**DOI:** 10.1038/s41392-022-00975-3

**Published:** 2022-04-13

**Authors:** BaoQing Chen, Mihnea P. Dragomir, Chen Yang, Qiaoqiao Li, David Horst, George A. Calin

**Affiliations:** 1 Department of Radiation Oncology, State Key Laboratory of Oncology in South China, Collaborative Innovation Center for Cancer Medicine, Sun Yat-sen University Cancer Center, Guangzhou, Guangdong, 510060 China; 2 Institute of Pathology, Charité-Universitätsmedizin Berlin, corporate member of Freie Universität Berlin, Humboldt-Universität zu Berlin and Berlin Institute of Health, Berlin, 10117 Germany; 3 Berlin Institute of Health, Anna-Louisa-Karsch-Straße 2, 10178 Berlin, Germany; 4 German Cancer Consortium (DKTK), Partner Site Berlin, German Cancer Research Center (DKFZ), 69210 Heidelberg, Germany; 5 Department of Translational Molecular Pathology, The University of Texas MD Anderson Cancer Center, Houston, TX 77030 USA; 6 Center for RNA Interference and Non-Coding RNAs, The University of Texas MD Anderson Cancer Center, Houston, TX 77030 USA

**Keywords:** Tumour biomarkers, Cancer genetics

## Abstract

It is now well known that non-coding RNAs (ncRNAs), rather than protein-coding transcripts, are the preponderant RNA transcripts. NcRNAs, particularly microRNAs (miRNAs), long non-coding RNAs (lncRNAs), and circular RNAs (circRNAs), are widely appreciated as pervasive regulators of multiple cancer hallmarks such as proliferation, apoptosis, invasion, metastasis, and genomic instability. Despite recent discoveries in cancer therapy, resistance to chemotherapy, radiotherapy, targeted therapy, and immunotherapy continue to be a major setback. Recent studies have shown that ncRNAs also play a major role in resistance to different cancer therapies by rewiring essential signaling pathways. In this review, we present the intricate mechanisms through which dysregulated ncRNAs control resistance to the four major types of cancer therapies. We will focus on the current clinical implications of ncRNAs as biomarkers to predict treatment response (intrinsic resistance) and to detect resistance to therapy after the start of treatment (acquired resistance). Furthermore, we will present the potential of targeting ncRNA to overcome cancer treatment resistance, and we will discuss the challenges of ncRNA-targeted therapy—especially the development of delivery systems.

## Introduction - overview of ncRNAs

The cataloging of the non-coding RNA (ncRNA) world is constantly and dramatically changing. Recent findings underscore the fact that a number of ncRNA transcripts can code for micropeptides (of less than 100 amino acids) that play functional roles in normal and pathological processes, including cancer.^1^ These novel data show that at least some ncRNAs have either both functional coding and non-coding capabilities or are, in fact, coding transcripts for non-classic peptides. Hence, we are facing the question of what ncRNA means.

**Fig. 1:**
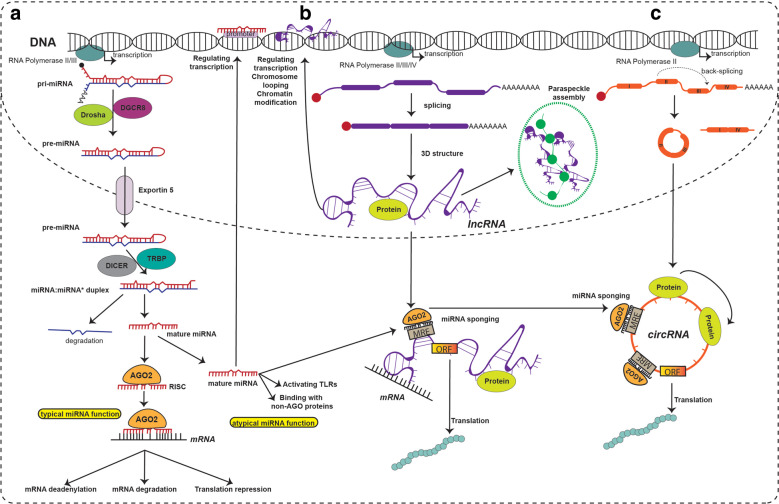
Biogenesis and function of miRNAs, lncRNAs and circRNAs. **a** *MIRNAs* are transcribed as primary miRNAs (pri-miRNAs) that contain the characteristic stem-loop structure. Pri-miRNAs are processed in the nucleus by Drosha and DGCR8 and transformed into precursor miRNAs (pre-miRNAs). Pre-miRNAs are transported from the nucleus to the cytoplasm via Exportin 5 and then are turned into an miRNA duplex after being cleaved by Dicer. One strand of the miRNA duplex is incorporated as part of the miRNA-induced silencing complex (RISC), and the second strand is degraded. By base-pairings between miRNAs and their target mRNA, the RISC binds an mRNA and suppresses its translation or induces its degradation. Additionally, there are unconventional/atypical miRNA functions such as activating Toll-like receptors (TLRs), binding non-AGO proteins, binding other ncRNAs (sponging), and regulating transcription. **b** Most lncRNAs have a biogenesis similar to mRNAs (although several exceptions exist), being capped, spliced, and adenylated. The mature lncRNAs adopt complex 3D structures that give them their multivalent functions. The function of lncRNAs can be divided according to their cellular localization: bound to chromatin (often *cis* functions), intranuclear (usually *trans* functions), and intracytoplasmic (*trans* functions). LncRNAs bound to chromatin usually function as regulators of transcription and induce chromosome looping and histone modifications. Nuclear lncRNAs can assemble paraspeckles and interact with nuclear proteins. Cytoplasmic lncRNAs bind mRNAs and act as decoys, guides, and scaffolds to transcriptionally or post-transcriptionally regulate downstream target genes, bind proteins to modify their function and stability, code for micropeptides that are being translated, and bind other ncRNA species (including miRNAs). **c** CircRNAs have multiple biogenesis mechanisms, but a common event for all is back-splicing. Back-splicing can be induced by protein dimerization, sequence complementarity of flanking introns, exon skipping mechanisms, and intron lariat debranching. After forming an uninterrupted RNA loop, the transcript is exported into the cytoplasm, where it serves as an miRNA sponge that inhibits miRNAs to regulate the expression of target genes, as a decoy of RNA-binding proteins to modulate gene expression or translation, or as a platform for protein-protein interaction; additionally, these transcripts also can be translated into micropeptides. As observed, there is direct crosstalk between lncRNAs and miRNAs and between circRNAs and miRNAs via sponging, creating a network of ncRNA molecules.

Currently, the most studied types of “classic ncRNAs” are microRNAs (miRNAs),^2^ long-non-coding RNAs (lncRNAs),^3^ and circular RNAs (circRNAs).^4^ MiRNAs are short RNAs that originate from longer stem-loop structures and can bind and inhibit mRNAs.^5^ The biogenesis of miRNAs is a multistep process. MiRNAs are transcribed as primary miRNAs (pri-miRNAs) and processed in the nucleus by Drosha and Dgcr8 into precursor miRNAs (pre-miRNAs). After they are exported to the cytoplasm, pre-miRNAs are cleaved to form an miRNA/miRNA duplex. Only one of the two miRNAs formed will exert its inhibitory function, the other one being degraded.^6^ Several other unconventional miRNA functions have been reported, including binding and inhibiting proteins, activating Toll-like receptors, coding for peptides, activating the translation of mRNAs, inhibiting mitochondrial transcripts, triggering transcription, and inhibiting nuclear ncRNAs,^7^ making miRNAs complex and versatile molecules (Fig. 1a). The total number of known human miRNAs is in continuous expansion and currently includes 1917 precursors and 2654 mature molecules (miRBase, release 22.1).^8^ Many additional miRNAs, mostly with tissue-specific distribution, have also been discovered.^9^ Most of these genes are conserved between species.^10^

MiRNAs’ role in cancer was revealed in 2002, when it was discovered that in chronic lymphocytic leukemia (CLL) *MIR15* and *MIR16* are frequently deleted and their transcripts downregulated.^11^ Currently, miRNAs have been reported to be dysregulated in each type of investigated cancer.^12–14^ The complexity of miRNAs’ mechanisms of action and the multitude of targets has made it difficult for researchers to translate their findings into clinical practice,^15^ and a better understanding of their role in oncology is necessary.

LncRNAs are by far the most complex type of ncRNAs, being arbitrarily defined as RNA molecules over 200 nucleotides long that are usually not translated into proteins. This definition is unfortunately vague. For example, because of their length, several primary miRNAs are considered to be lncRNAs if they have a function on their own, and of all ncRNAs, lncRNAs have the highest potential of coding peptides (this has been confirmed several times).^1^ The biogenesis of lncRNAs is similar to that of mRNAs, many of them being spliced, capped, and poly-adenylated. The complexity of these transcripts comes from their multifaceted 3D structure, which rapidly changes and gives them the ability to perform multiple functions.^16,17^ LncRNAs have *cis* (performed in the proximity of their transcription site) and *trans* (performed distant from the transcription site) functions.^18^ Typical *cis* functions are related to DNA transcription, chromatin modifications, and chromosomal looping. Well-defined *trans* functions include binding to mRNAs and changing their stability, binding to proteins and altering their function, interacting with other ncRNAs, and facilitating the assembly of paraspeckles^19,20^ (Fig. 1b). The research on lncRNA surged after the 2003 discovery of *MALAT1*’s involvement in the metastasis of non-small cell lung cancer (NSCLC).^21^ Similar to miRNAs, lncRNAs’ role in cancer is now well studied but only rarely translated into clinical practice, the exception being *PCA3* as a biomarker for prostate cancer.^22^

A complementary approach is to classify lncRNA from a phylogenetic standpoint. LncRNAs can be transcripts of ultraconserved elements that are identical in mice, rats, and humans.^23^ These lncRNAs are named transcribed-ultraconserved regions and because of their high degree of conservation are expected to have essential functions.^24^ On the other hand, are the more recently emerged transcripts, the primate-specific lncRNAs, which often contain transcribed pyknons in their structure.^25,26^ Pyknons are short, primate-specific, repetitive DNA motifs that are often localized in DNA-fragile sites and are transcribed as part of lncRNAs.^25,27^ These lncRNAs containing pyknons have low expression levels in normal cells; however, their expression level spikes in malignant and immune cells, making them ideal candidates for future therapies.^20,28^

CircRNAs, the third major class of ncRNAs, are characterized by their specific structure. CircRNAs are covalently closed uninterrupted loops, where the 3’ and 5’ ends are joined together.^29^ Because of this structure, circRNAs are more stable than other RNA types.^29^ CircRNAs have a complex and multifaceted biogenesis, for which multiple mechanisms have been described over the past years. CircRNAs can be generated by exon skipping mechanisms, intron lariat debranching, intron pairing, and RNA binding proteins dimerization.^30^ The functions of circRNAs are only partially characterized. CircRNAs have been described as super-spongers, being able to bind tens of miRNA molecules and inhibit their function.^31,32^ However, only a few circRNAs are capable of binding multiple miRNA molecules.^29^ Similar to lncRNAs and immature miRNAs, circRNAs can code for micropeptides.^1^ Additionally, circRNAs bind proteins^33^ and regulate their functions and can control translation^29^ (Fig. 1c). These functions are seen more as exceptions than rules, and the mechanistic roles of circRNAs need to be further researched. Their role in cancer was initially revealed through deep sequencing profiling when it was observed, in 2013, that many circular transcripts are abundant and differently expressed in multiple cancer cell lines.^34^ Soon after, this observation was confirmed in patients’ samples.^29^

The three ncRNA classes have been extensively linked to different malignant processes, including resistance to various cancer therapies. Interesting is the fact that the same miRNA was shown to be an oncogene in one cancer and a tumor suppressor gene in another cancer.^6^ Hence, miRNAs play a context-dependent role in tumorigenesis. LncRNAs are known to regulate all hallmarks of cancer, and because of their 3D structure, single nucleotide polymorphisms and mutations can induce important functional switches that have only recently started to be characterized.^35^ CircRNAs are the “newest” addition and have also been linked to all cancer hallmarks, their function in cancer being mainly explained by miRNA sponging.^29^ Indeed, all three classes of ncRNA directly or indirectly interact—lncRNAs and circRNAs can bind miRNAs and inhibit their binding to mRNAs—so a complex network of RNA molecules exists. In order to discover crucial targets that could reverse therapy resistance in cancer, this network’s essential hubs need to be revealed.

In recent years, we have witnessed multiple high-throughput studies (e.g., genome sequencing, transcriptomics, proteomics) researching the role of mutational, transcriptional, and translational aberrations in drug resistance.^36^ Nevertheless, a thorough understanding for lack of response to therapy in many instances has not yet been found. We suggest that the constantly increasing number of ncRNAs—which includes other species such as transfer RNAs (tRNAs), ribosomal RNAs (rRNAs), piwi-interacting RNA (piRNAs), small nuclear RNA (snRNAs), and small nucleolar RNAs (snoRNAs), not discussed here for the lack of space and because they are beyond the scope of this paper, but reviewed by others^37^—could be the missing elements needed to understand therapy resistance. First, ncRNA levels change quickly and are extremely heterogeneous between tumors with similar histological subtypes. This makes ncRNAs difficult to use as screening and diagnostic biomarkers but interesting biomarkers for sub-classifying a tumor type and hence useful tools for personalized medicine. These quick changes in expression (bursts) that we observe, which in many cases are from undetectable (i.e., not expressed) to highly expressed, can explain the phenomenon of acquired resistance—which sometimes takes place quickly and is hard to understand in the context of slow events having a complex mechanism of occurrence, such as mutations, translational changes, or epigenetic alterations. Second, ncRNAs are extremely versatile. The three classes of ncRNAs have multiple functions, and the phenomenon of resistance can emerge not by changes in transcription level but by changes in function. “Functional switches” are not well studied in the context of therapy resistance but are well documented in various pathological mechanisms for all three types of ncRNAs. The best studied functional switches are those for miRNAs; for example, miR-21-5p can bind TLR8 and induce a protumorigenic inflammatory response,^38^ and pri-miR-200a and -200b can be translated into micropeptides that inhibit epithelial-mesenchymal transition.^6^ This shows that in some instances, no change in expression is necessary for inducing phenotypical changes, but only a functional switch. Functional switches are most probably dependent of the subcellular localization of ncRNAs, and we believe that a better understanding of such mechanisms will be achieved with the development of spatial transcriptomics for ncRNAs. Finally, as we already mentioned, there is a complex interplay between the different classes of ncRNAs as each type of ncRNA can bind any other type, creating intricate networks,^39,40^ and a change in one ncRNA can induce a domino effect that can modify a vast number of molecules.

Hence, we consider ncRNAs to be potential markers that can predict a personalized response to therapy or even adjuvants that can increase response to conventional therapy. In the next section, we will present some prominent examples of ncRNAs that play important roles in therapy resistance.

## Mechanisms of therapy resistance mediated by ncRNAs

Treatment resistance^41^ can be classified as intrinsic or acquired according to the timepoint when the resistance develops. Intrinsic resistance is the innate resistance that exists before the initiation of treatment or develops within a short duration after treatment initiation. Intrinsic resistance represents a lack of response to the initial treatment. Acquired resistance occurs after a certain duration of the treatment.^42^ In this scenario, the cancer initially responded to treatment but later progressed.

Intrinsic resistance usually is caused by the following mechanisms: (1) innate genetic aberrations leading to the poor response to various cancer therapies, e.g., NSCLC with EGFR (epidermal growth factor receptor) T790M de novo mutation has no response to first- and second-generation EGFR tyrosine kinase inhibitors (TKIs)^43^ and breast cancer with absence of estrogen receptors or progesterone receptor does not benefit from endocrine therapy;^44^ (2) heterogeneity within tumors tissues in which pre-existing resistant subpopulations will survive anti-cancer treatment, e.g., cancer stem cells with the capacity of self-renewal and differentiation will survive and contribute to tumor repopulation and growth;^45^ (3) protection induced by the activation of defending intrinsic pathways against xenobiotics, e.g., activation of ATP-binding cassette efflux transporters or the glutathione/glutathione S-transferase system to cause the efflux of chemotherapeutic drugs.^46^

There are also multiple mechanisms of developing acquired resistance: (1) driver oncogene modification, e.g., development of EGFR T790M mutation, but not de novo alteration, is observed within 1 year in about 50% of NSCLC patients treated with the first and second generations of TKIs, resulting in tumor progression;^43^ (2) activation of independent pro-survival parallel signaling, e.g., cell proliferation, apoptosis, or autophagy and cell metabolism signaling;^47^ (3) adaption of the tumor microenvironment after the start of treatment. Of note, these mechanisms of developing the intrinsic and acquired resistance usually co-exist and contribute to tumor progression; thus, it is more practical to understand the exact underlying mechanisms of resistance development than to seek insight into intrinsic and acquired resistance separately. NcRNAs directly or indirectly modulate the treatment sensitivity by finely orchestrating these underlying mechanisms. Overexpression of some lncRNAs can function as tumor driver oncogenes to promote the intrinsic chemotherapy resistance, while others are overexpressed after the induction of treatment and then modulate survival signaling to promote tumor repopulation, leading to acquired resistance. This section will give a short outline of the roles of the most studied ncRNAs in intrinsic or acquired therapeutic resistance and their potential mechanisms.

### Resistance to chemotherapy

Many factors can induce chemotherapy resistance, but probably the most important is tumor heterogeneity.^48^ For intrinsic resistance, intertumoral heterogeneity plays a crucial role, and the genetic variability (germline variations) between patients harboring neoplasia of the same histotype explains why only some tumors will respond to a given chemotherapy agent. For acquired resistance, spatial and temporal intratumoral heterogeneity is the key element,^48^ and it is accepted that chemotherapy induces the selection of tumor cell populations that are resistant. One of the crucial mechanisms behind intratumoral heterogeneity is chromosomal instability (CIN), the continuous duplications and deletions of chromosomal regions during cancer cell division (Box 1).

For almost a decade, it was known that *CCAT2*, an lncRNA located in the frequently amplified 8q24 region, is overexpressed in colorectal cancer (CRC) and is associated with CIN.^49^ Recently, the molecular mechanism related to its role in CIN was revealed. *CCAT2* binds BOP1 and AURKB, two proteins known to be associated with CIN, and increases the number of chromosomal aberrations. As expected, this induces abnormal mitosis in vitro and in vivo. Not surprisingly, high *CCAT2* levels in CRC cell lines are associated with resistance to the two main chemotherapeutics used in gastrointestinal cancers, 5-flurouracil (5-FU) and oxaliplatin.^50^ By studying the role of mesenchymal stem cells (MSCs) in gastric cancer, He et al. proved that their role in chemoresistance is mediated by the lncRNA *MACC1-AS1*. The researchers discovered that MSCs induce stemness and chemotherapy resistance by secreting transforming growth factor β1, which in gastric cancer cell lines induces overexpression of SMAD2 and SMAD3, which in turn activate *MACC1-AS1* expression. *MACC1-AS1* binds and inhibits miR-145-5p, derepressing to key elements (CPT1 and ACS) of the fatty acid oxidation pathway. In vivo experiments reveled that inhibition of the fatty acid oxidation pathway restored gastric cancer sensitivity to the FOLFOX regimen, which includes 5-FU, oxaliplatin, and folinic acid.^51^

By comparing cisplatin-resistant with cisplatin-sensitive bladder cancer cell lines, Drayton et al. detected a signature of dysregulated miRNAs that is associated with resistance development. To better characterize the resistance mechanism, the authors analyzed whether the resistance is mediated by cellular metabolic changes prior to DNA adduct formation or via DNA damage repair mechanism after adduct formation. Unexpectedly, they observed that cisplatin resistance in bladder cancer is induced by an altered cisplatin metabolism in which production of glutathione and SLC7A11 are increased. It is important to mention that intracellular glutathione binds cisplatin and detoxifies the intracellular environment. One of the miRNAs found downregulated in the initial screening, miR-27a, directly binds SLC7A11 and decreases glutathione production. Hence, low levels of miR-27a are responsible for cisplatin resistance in bladder cancer. Finally, in clinical samples, the authors confirmed that high levels of SLC7A11 and low levels of miR-27a are associated with poor prognosis.^52^

In an attempt to understand the mechanism of cisplatin resistance in gastric cancer, it was observed that patients who acquired resistance had a significantly higher level of circAKT3, and high circAKT3 was associated with shorter overall survival. Indirectly, it was observed that high levels of circAKT3 increase the level of genomic instability by interfering with the DNA damage repair protein BRCA1. Additionally, circAKT3 inhibits the function of miR-198, which depresses the oncoprotein PIK3R1, which in turn activates the well studied PI3K/AKT oncogenic pathway.^53^

Box 1 Cancer therapy, chromosomal instability and lncRNAs Chromosomal instability (CIN) was the first hallmark of cancer to be discovered, and the history of CIN is probably a little older than most researchers know. More than a decade before Theodor Bovari and Walter Sutton postulated their theory that chromosomal aberrations cause cancer, Leo Hansemann made the first drawings of aberrant mitosis in cancer.^169^ Unfortunately, Hansemann never came up with a biological interpretation for his observations. Bovari was most probably inspired by Hansemann’s drawings and mentioned them several times.^169^ No evidence exists that Sutton consulted Hansemann’s drawings. Not surprisingly, CIN is one of the catalysts that induces acquired resistance to radio- and chemotherapy by continuously generating heterogeneous cell populations that eventually do not respond to treatment.^170^ We recently showed that the lncRNA *CCAT2*, which is highly overexpressed in colorectal cancer^49^ and myeloproliferative neoplasia, and can induce myeloproliferative neoplasia in vivo,^171^ is a component of the CIN pathway. In a mechanistic study, we revealed that *CCAT2* is a master regulator of CIN. *CCAT2*, BOP1, and AURKB form an RNA-protein complex^50^ that pulls the chromosomes sketched by Hansemann over 100 years ago in all directions, creating chaos in cancer cell division. Other lncRNAs have also been linked to CIN; for example, it was shown that the lncRNA *NORAD* preserves normal mitosis by binding and inhibiting PUMILIO proteins that, if hyperactivated, can induce CIN.^172^ There are two possible therapeutic strategies to restore radio- and chemotherapy response via the CIN pathway. The cancer cell cannot tolerate too much CIN; hence, one can accelerate CIN pathways and generate less-fit karyotypes. The other option is to inhibit CIN and therapeutically tackle a stable and genetically frozen cancer cell population. We believe that by overexpressing/inhibiting CIN-associated lncRNAs, CIN-induced resistance to therapy can be manipulated.

### Resistance to targeted therapy

Targeted therapy development was possible due to the evolution from an empirical-based drug discovery approach to a rational approach in which an aberrant dominant mutation, gene amplification, or oncogenic translocation that drives tumor growth is targeted.^54^ One characteristic of targeted therapy, especially for solid tumors, is that only a minority of tumors rely on the hyperactivation of the targeted genes to evolve.^54^ In patients with intrinsic resistance, targeted therapy will not be started because molecular analysis shows that the drivers are missing. In patients who are candidates for targeted therapy, response is usually not permanent but temporary. After the initial response phase, acquired resistance develops.

A commonly used targeted therapeutic agent, sunitinib, is a TKI approved for the treatment of gastrointestinal stromal tumors (GISTs), pancreatic neuroendocrine tumors, and renal cell carcinomas (RCCs). Unfortunately, up to 20% of patients with RCC show an intrinsic resistance to sunitinib, and most of the other patients develop resistance during the course of therapy.^55^ Qu et al. used in vitro and in vivo screening algorithms to discover new pathways associated with sunitinib resistance.^56^ They observed that a previously uncharacterized lncRNA, *lncARSR* (lncRNA activated in RCC with sunitinib resistance), is upregulated after resistance development. Using multiple clinical samples, they observed that the level of circulating *lncARSR* in plasma was higher in patients with progressive disease and that high levels were associated with shorter overall survival. Mechanistically, it was observed that the RNA binding protein hnRNPA2B1 packs *lncARSR* into exosomes, and these are transferred between cells, disseminating sunitinib resistance. Moreover, by injecting exosomes from sunitinib-resistant cells into naïve tumors of mice, they induced sunitinib resistance in vivo. They showed that at the intracellular level, *lncARSR* binds miR-34a and miR-449, indirectly upregulating AXL and c-MET. Finally, in a proof-of-concept experiment, the authors restored sunitinib resistance in vivo by targeting *lncARSR* using a complementary locked nucleic acid inhibitor.^56^

After establishing a 3D model of resistance to the EGFR inhibitor cetuximab, Lu et al. discovered that the most notable transcriptional event acquired by the newly developed model was an upregulation of *MIR100HG* primary transcript and the two mature hosted miRNAs, miR-100 and miR-125b. Phenotypically, the two miRNAs additively play an oncogenic role and mediate cetuximab resistance in vitro and in vivo. Mechanistically, miR-100 and miR-125b inhibit five negative regulators of the Wnt signaling pathway: DKK1, DKK3, ZNRF3, RNF43, and APC2, hence stimulating this pro-oncogenic circuit. The upstream expression of the lncRNA *MIR100HG* is negatively regulated by the GATA6 transcription factor, which is downregulated in cetuximab-resistant and advanced stage CRC. Moreover, miR-125b binds the 3’UTR of *GATA6*, inducing its post-transcriptional inhibition and creating a double negative feedback circuit. Clinical data showed an important increase in *MIR100HG* and its embedded miRNAs and a decrease in GATA6 at the time of disease progression during cetuximab treatment.^57^

Sorafenib is a multi-kinase inhibitor approved for the treatment of advanced RCC, hepatocellular carcinoma (HCC), and thyroid cancers. A significant number of patients with HCC respond poorly to sorafenib, while responders frequently develop resistance during the first 6 months of therapy.^58^ Starting from the observation that high miR-541 levels are associated with longer overall survival in HCC, Xu et al. study the anti-oncogenic function of this miRNA. miR-541 directly targets *Ras-related protein RAB1B* and *autophagy-related gene 2* *A* (*ATG2A*), strongly inhibiting autophagy both in vitro and in vivo. More remarkable is the fact that high levels of miR-541, in an additive manner, potentiate the anti-tumorigenic effect of sorafenib. This phenomenon is most probably mediated via inhibiting RAB1B and ATG2A. Clinical data strongly support these findings; patients with a high level of miR-541 who were treated with sorafenib had significantly longer survival compared to patients with high miR-541 and without sorafenib therapy.^59^ Another study by Xu et al. showed that circRNAs also can influence resistance to sorafenib. CircRNA-SORE (a circRNA upregulated in sorafenib-resistant HCC cells) not only is upregulated in multiple sorafenib-resistant cell lines but is a key element in maintaining that resistance. At the molecular level, circRNA-SORE directly binds in the cytoplasm the oncogenic protein YBX1 and prolongs its half-life by blocking its transfer into the nucleus where it is degraded by PRP19. Similar to *lncARSR*, circRNA-SORE is transferred from resistant cells to naïve cells via exosomes and induces a widespread resistance to sorafenib. By treating mice bearing subcutaneous sorafenib-resistant patient-derived xenograft tumors with small interfering RNA (siRNA) against circRNA-SORE, the authors showed that inhibition of the circRNA can restore sorafenib resistance.^60^

### Resistance to radiotherapy

It is accepted that radioresistance is controlled by intrinsic factors arising from tumor cells, mainly the genomic instability characteristic for many neoplasia,^61^ or by extrinsic factors represented by multiple components of the tumor microenvironment (i.e., the immune component, vascular component, and pro-fibrotic stromal component).^62^

Starting from the observation that *linc00312* is downregulated in nasopharyngeal carcinoma compared to chronic rhinitis, Guo et al. studied its role in cancer. They discovered that this lncRNA is much higher in radiotherapy-treated patients with complete response compared to those with partial response and progressive disease/radioresistance. In vitro experiments confirmed the tumor suppressor function of nuclear *linc00312*, which inhibits proliferation, activates apoptosis, and renders radiosensitivity to cancer cells. At a molecular level, *linc00312* directly binds the catalytic subunit of DNA-dependent protein kinase, inhibiting its interaction with the Ku80 subunit after DNA double-strand breaks. Hence, it seems that *linc00312* potentiates radiotherapy by blocking the DNA repair machinery.^63^

By comparing patients with breast cancer whose disease relapsed after radiotherapy versus those whose disease did not relapse, it was observed that a panel of miRNAs is dysregulated. In particular, miR-139-5p was downregulated in patients with unfavorable outcomes, and its overexpression was associated with high sensitivity to radiotherapy in vitro. Mechanistically, it was observed that this miRNA targets multiple genes with important roles in DNA repair and reactive oxygen species (ROS) defense, including MAT2A, POLQ, TOP1, and TOP2A. By overexpressing miR-139-5p in radiotherapy-resistant cells, the DNA repair mechanism was blocked and apoptosis induced. Using a massive patient cohort, it was confirmed that high levels of miR-139-5p and low levels of POLQ, TOP1, and RAD54L are associated with better survival, but only in radiotherapy-treated patients. Finally, by using miR-139-5p mimetics in a proof-of-concept experiment in vivo, it was proven that miR-139-5p is a potent radiotherapy sensitizer.^64^

Yuan et al. discovered that high levels of miR-410 induce radiotherapy resistance in NSCLC by accelerating DNA damage repair. At the molecular level, miR-410 directly binds and inhibits the translation of the tumor suppressor *PTEN*, which in turn activates the PI3K/mTOR signaling pathway. Moreover, miR-410 also activates epithelial-mesenchymal transition (EMT) via the PI3K/mTOR signaling pathway. Clinical observations confirmed these findings: miR-410 is overexpressed in EMT and mesenchymal tumors and is associated with low levels of PTEN.^65^

The paradigm regarding the meaning of non-coding is shifting. Recently it was shown that in glioblastoma multiforme, the levels of the already mentioned circAKT3 drop. But much more surprising, this circRNA encodes protein AKT3-174aa, which is 174 amino acids long and plays important anti-tumorigenic roles. AKT3-174aa interacts with the RTK/PI3K/AKT pathway, inhibiting the phosphorylation of AKT at Thr308. From a therapeutic standpoint, AKT3-174aa overexpression restored glioblastoma cells’ sensitivity to radiotherapy. Therefore, we can envision in the near future the delivery of ectopic proteins/peptides encoded by ncRNAs as new adjuvants to restore sensitivity to radiotherapy.^66^

### Resistance to immune checkpoint inhibitors

Immune checkpoint inhibitors (ICIs), monoclonal antibodies directed against immune checkpoint molecules such as PD-1, PD-L1, and CTLA-4, are the newest addition to cancer therapy. These drugs are true game changers of cancer therapy, inducing durable disease control and prolonged response. Unfortunately, not all treated patients experience effective responses.^67^ Mechanisms of resistance to immune checkpoint therapy can be divided into (1) deficient anti-tumor T cell production, (2) poor anti-tumor T cell effector function, and (3) impaired development of T cell memory.^68^ Additionally, resistance to ICIs was linked to other immune cells such as natural killer (NK) cells and myeloid-derived suppressor cells (MDSCs).

Starting from the observation that the lncRNA *LINK-A* is overexpressed in patients whose disease does not respond to pembrolizumab and has a negative correlation with CD8^+^ T lymphocyte and antigen-presenting cell expression, Hu et al. described the function of this lncRNA in the intrinsic resistance to ICI. The authors used an existing breast cancer mouse model in which they overexpressed *LINK-A* and discovered that it induces an aggressive triple-negative breast cancer phenotype that metastasizes to the lungs. Mechanistically, *LINK-A* facilitates the interaction between phosphatidylinositol-(3,4,5)-trisphosphate (PtdIns(3,4,5)P_3_) and G-protein–coupled receptor, decreasing the phosphorylation of TRIM71. An outcome of this interaction leads to increased degradation of TP53, Rb, and the antigen peptide-loading complex. Furthermore, this molecular cascade decreases the number of CD8^+^ T cells and granzyme B NK cells in the peritumoral milieu.^69^

An additional element associated with ICI resistance is MDSCs, high levels of which may be associated with resistance to ICIs.^70^ Huber et al. discovered that multiple miRNAs, miR-146a, miR-155, miR-125b, miR-100, let-7e, miR-125a, miR-146b, and miR-99b, are released by melanoma cells via extracellular vesicles (EVs). Consequently, EVs containing this set of miRNAs are internalized into myeloid cells, which in turn acquire an MDSC phenotype. Clinical data revealed that in patients with stage IV melanoma treated with the ICIs nivolumab or ipilimumab, high levels of this set of circulating miRNAs are associated with shorter overall survival.^71^ Hence, we can envision combining ncRNA therapy with ICIs to overcome resistance (Box 2).

Huang et al. adopted a classic method to study therapy resistance in HCC; they started by analyzing genes located in the 7q21-7q31 amplicon associated with an unfavorable outcome. They observed that circMET is located in this region, is overexpressed in HCC, and is associated with unfavorable outcomes. At a phenotypical level, they noticed that circMET overexpression induces EMT and potentiates the immunosuppressive tumor microenvironment. Immunologically, circMET decreases the density of CD8^+^ lymphocytes in tumor tissue. At the molecular level, circMET sponges miR-30-5p and indirectly upregulates the transcription factor Snail. Snail activates the expression of DPP4, which in turn inhibits the chemotactic molecule CXCL10, hence blocking CD8^+^ immune cell trafficking. Finally, in vivo studies showed that if this axis is activated, anti-PD-1 therapy resistance emerges.^72^

**Fig. 2:**
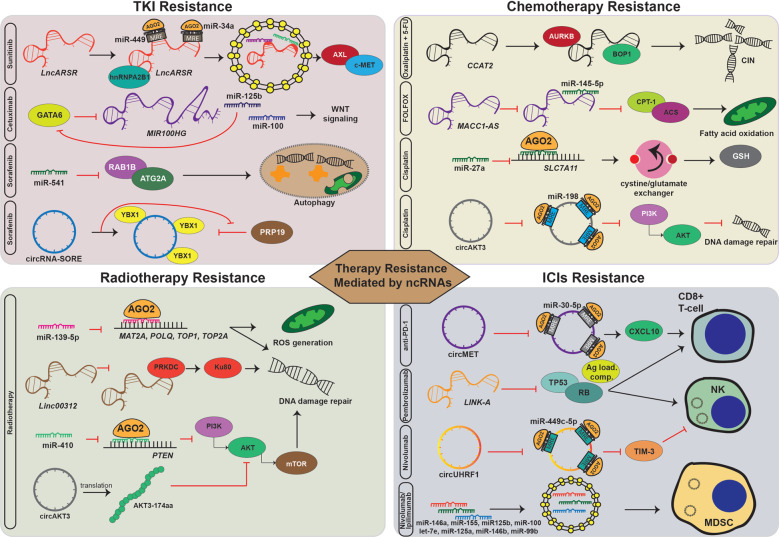
Mechanisms of therapy resistance mediated by ncRNAs. Examples of the common mechanisms of cancer cell resistance to tyrosine kinase inhibitors, chemotherapy, radiation, and immune checkpoint inhibitors mediated by miRNAs, lncRNAs, or circRNAs. The common mechanisms include (1) modulation of defending intrinsic pathways against the xenobiotics, e.g., miR-27a directly binds to SLC7A11 and decreases the glutathione (GSH), which binds cisplatin and detoxifies the intracellular environment, thus a decrease of miR-27a is responsible for cisplatin resistance; (2) promoting survival signaling pathways, e.g., *lncARSR*, which is packed by hnRNPA2B1 and then binds *to* miR-34a and miR-449, indirectly upregulates AXL and c-MET to contribute to sunitinib resistance; *MIR100HG* and its embedded miRNAs, miR-100 and miR-125b, mediate cetuximab resistance by activating Wnt signaling; circRNA-SORE directly binds to oncogenic protein YBX1 and prolongs its half-life by blocking its transfer into the nucleus, where it is degraded by PRP19 to trigger sorafenib resistance; (3) accelerating DNA damage repair, e.g., miR-410 inhibits the translation of *PTEN*, leading to the activation of the PI3K/mTOR signaling and accelerating DNA damage repair to induce radiotherapy resistance; circAKT3 inhibits miR-198, which in turn activates the PI3K/AKT signaling and triggers cisplatin resistance; (4) inducing genomic instability, e.g., lncRNA *CCAT2* binds with BOP1 and AURKB to induce chromosomal instability (CIN) and resistance to 5-flurouracil (5-FU) and oxaliplatin; (5) inhibition of cell apoptosis or autophagy, e.g., miR-541 targets *Ras-related protein RAB1B* and *autophagy-related gene 2* *A* (*ATG2A*), inhibiting autophagy, and further accelerates sorafenib resistance; (6) regulating cell metabolism, e.g., *MACC1-AS1* binds and inhibits miR-145-5p, derepressing to key elements (CPT1 and ACS) of the fatty acid oxidation pathway, leading to resistance to the FOLFOX chemotherapy regimen; and (7) tuning the infiltrated immune cells, including T cells, myeloid-derived suppressor cells (MDSCs), and natural killer cells in the tumor immune microenvironment, e.g., circMET sponges miR-30-5p and indirectly inhibits the chemotactic molecule CXCL10, hence blocking CD8^+^ immune cell trafficking; *LINK-A* facilitates the degradation of TP53 and Rb, thus decreasing the number of CD8^+^ T cells and granzyme B NK cells; circUHRF1 binds and inhibits miR-449c-5p, upregulating TIM-3, to inhibit NK cell function; and miR-146a, miR-155, miR-125b, miR-100, let-7e, miR-125a, miR-146b, and miR-99b are released by melanoma cells via extracellular vesicles and internalized into myeloid cells to drive MDSC differentiation.

An analysis of the role of another circRNA, circular ubiquitin-like with PHD and ring finger domain 1 RNA (circUHRF1), in anti-PD-1 resistance in HCC showed that NK cells also play an important role. Like circMET, circUHRF1 is overexpressed in HCC, and high levels are associated with advanced T category, decreased circulating NK cells, microvascular invasion, and short overall and relapse-free survival after surgery. Interestingly, circUHRF1 is secreted into exosomes by HCC cells, and its plasma levels are much higher before surgery and during relapse compared to after surgery or in healthy controls. At the immunological level, exosomal circUHRF1 derived from HCC cells inhibits NK cell function. In NK cells, circUHRF1 binds and inhibits the biological function of miR-449c-5p and indirectly upregulates the expression of the immune checkpoint–T cell immunoglobulin and mucin domain-containing protein 3 (TIM-3). Further clinical analysis revealed that high circUHRF1 expression is associated with progressive disease in HCC patients treated with anti-PD-1 and negatively correlates with NK cells in tumor tissue. In vivo studies confirmed the results: mice treated with anti-PD-1 treatment plus circUHRF1 shRNA have significantly longer overall survival compared to mice treated only with anti-PD-1.^73^ Whether circUHRF1-mediated resistance to anti-PD-1 therapy is intrinsic or acquired needs to be further analyzed. An overview of the role of ncRNAs in therapy resistance can be found in Table 1 and Fig. 2.

**Table 1:** A compendium of ncRNA-related targets and mechanisms in resistance to targeted therapy, chemotherapy, radiotherapy, and immunotherapy

Human ncRNA	Expression: down- or upregulated; cancer type	Function in therapy resistance	Molecular mechanisms and targets	Ref
*Resistance to chemotherapy*				
*CCAT2*	Upregulated in MSS (CIN) CRC	High levels are associated with resistance to 5-fluorouracil and oxaliplatin	Binds to BOP1 and AURKB, increasing aberrant mitosis and abnormal karyotypes	^50^
*MACC1-AS1*	Overexpressed in FOLFOX-resistant GC	High levels activate fatty acid oxidation pathway	Binds miR-145-5p, derepressing CPT1 and ACS	^51^
MiR-27a	Downregulated in cisplatin-resistant bladder cancer	Low levels are associated with increased glutathione production that detoxifies the intracellular milieu	Binds SLC7A11 and inhibits its translation	^52^
CircAKT3	Upregulated in cisplatin-resistant gastric cancer	High levels are associated with cisplatin resistance and increased DNA damage	Binds miR-198, derepressing PIK3R1 and consequentially activating the PI3K/AKT signaling pathway	^53^
*Resistance to targeted therapy*				
*LncARSR*	Upregulated in tissue and plasma of renal cell carcinoma patients with sunitinib resistance	Induces sunitinib residence that is transferable between cells	Binds miR-449 and miR-34a, indirectly upregulating AXL and c-MET	^56^
*MIR100HG* (miR-100, miR125b)	Upregulated in CRC and HNSCC cells and CRC patients at time of progression on cetuximab	Activated at time of progression on cetuximab	The two miRNAs activate the Wnt signaling pathway by inhibiting five of its negative regulators: DKK1, DKK3, ZNRF3, RNF43, and APC	^57^
MiR-541	Upregulated in HCC with long overall survival, high in HCC patients who responded to sorafenib	High levels in an additive manner increase the anti-tumorigenic effect of sorafenib	Inhibits autophagy via directly inhibiting RAB1B and ATG2A	^59^
CircRNA-SORE	Upregulated in HCC, higher in patients treated with sorafenib who had short RFS	Induces sorafenib residence that is transferable between cells	Binds to and prolongs the half-life of the oncoprotein YBX1 by preventing its degradation	^60^
*Resistance to radiotherapy*				
*Linc00312*	Overexpressed in chronic rhinitis versus nasopharyngeal carcinoma	Blocks the DNA double-stand break repair machinery	Directly binds DNA-PKcs and inhibits its interaction with Ku80	^63^
MiR-410	High levels are associated with radioresistance in NSCLC	Enhances the DNA damage repair machinery upon irradiation	Directly binds and inhibits PTEN, indirectly activating the PI3K/mTOR pathway	^65^
MiR-139-5p	Downregulated in breast cancer patients who experience relapse after radiotherapy	High levels of miR-139-5p inhibit DNA repair genes and ROS defense mechanism	Directly binds and inhibits the translation of MAT2A, POLQ, TOP1, and TOP2A	^64^
CircAKT3	Downregulated in GBM	Low levels are associated with radioresistance	Encodes for a 174 aa protein, which inhibits the phosphorylation of AKT at Thr308	^66^
*Resistance to immunotherapy*				
*LINK-A*	Upregulated in TNBC patients who do not respond to pembrolizumab	Decreases the number of tumor-infiltrating CD8^+^ and NK cells	Induces degradation of the antigen peptide-loading complex, TP53 and Rb.	^69^
MiR-146a, miR-155, miR-125b, miR-100, let-7e, miR-125a, miR-146b, and miR-99b	Upregulated in plasma EVs from advanced stage melanoma patients	Increase the number of tumor-infiltrating myeloid cells	Induce the transformation of monocytes into MDSCs	^71^
CircMET	Upregulated in HCC, associated with unfavorable outcomes	Induces anti-PD-1 residence by decreasing intratumoral CD8^+^ cells	Sponges miR-30-5p, derepressing Snail, which indirectly inhibits CXCL10 via DPP4	^72^
CircUHRF1	Upregulated in HCC, associated with unfavorable response to anti-PD-1 therapy	Induces anti-PD-1 residence by suppressing NK cell function	Sponges miR-449c-5p, indirectly activating the immune checkpoint molecule TIM-3	^73^

*CIN* Chromosomal instability; *CRC* Colorectal cancer; *DNA-PKcs* DNA-dependent protein kinase catalytic subunit; *EV* Extracellular vesicles; *FOLFOX* Folinic acid, fluorouracil, and oxaliplatin; *GC* Gastric cancer; *GBM* Glioblastoma multiforme; *HCC* Hepatocellular carcinoma; *HNSCC* Head and neck squamous carcinoma; *MDSC* Myeloid-derived suppressor cells; *MSS* Microsatellite stable; *NSCLC* Non-small cell lung cancer; *RFS* Recurrence-free survival; *TNBC* Triple-negative breast cancer.

Box 2 Combining ncRNA therapeutics with immune checkpoint inhibitors Several ncRNAs were found to be involved in the immune checkpoint–mediated cancer cell mechanism for evading immune destruction. For example, several miRNAs are known to directly and indirectly regulate the expression of immune checkpoint molecules, not only the canonical ones (CTLA-4, PD-1, and PD-L1) but also the less studied ones such as B7-H3, BTLA, TIM-3, and LAG-3.^125^ Hence, we can envision, similar to combined immune checkpoint inhibitor (ICI) therapy, a strategy to overexpress these miRNAs in combination with ICIs to block an alternate pathway of immunotolerance induced by immune checkpoints. Such a strategy might also reduce the unwanted side effects of combined ICI therapy, which are worse than those induced by monotherapy.^173^ Moreover, some miRNAs target multiple immune checkpoints. One such example is miR-138, which can directly target CTLA-4, PD-1, and PD-L1.^174,175^ CircRNAs regulate the expression of immune checkpoints indirectly, usually via miRNAs, by inhibiting their function. For example, the same miR-138, in colorectal cancer, is sponged by hsa_circ_0020397, leading to the overexpression of PD-L1.^175^ Therefore, a complex network containing multiple species of ncRNAs regulates the expression of immune checkpoints, providing multiple targets that can be used to manipulate response to immunotherapy. LncRNAs play much more intricate roles, and their mechanistic interrelation with immune checkpoints is only scarcely described. Their role as potential co-therapeutics with ICIs was recently demonstrated. The lncRNA *UCA1* and PD-1 were knocked out in mouse tumors, and the combined knockout decreased the tumor burden and prolonged overall survival by modulating the T cell–mediated immune response.^176^ These data together prove the valuable role ncRNA modulation can play in ICI therapy.

## Non-coding RNAs as biomarkers for therapy resistance

Biomarkers are regarded as signs of a biological process, indicating a certain condition or disease, and are usually assessed, invasively or non-invasively, from body fluids or tissues.^74^ A feasible cancer biomarker is the one that is expressed by a specific type of cancer cell, differentially expressed compared to normal tissue, or dynamically altered during cancer progression or the course of treatment.^75^ Such biomarkers have assumed a growing role in distinguishing malignant from benign disease, predicting patient prognosis, monitoring cancer recurrence, and determining response to anti-cancer therapy. Prominent biomarker candidates are identified as proteins (i.e., cytokines and receptors) and nucleic acids (i.e., DNA, RNA), including ncRNAs.^76^ Vast evidence reveals that some ncRNAs are the preferential biomarker in the diagnosis of certain cancers, especially when comprehensively combined with other biomarkers.^77,78^ Tissue-specific, cell-specific, and developmental stage–specific expression patterns give ncRNAs great value as clinical biomarkers in certain cells, tissues, and conditions. By annotating the gene expression of 16 tissues through GENCODE consortium, the expression patterns of 14,880 lncRNAs were revealed. Compared to protein-coding genes, 65% of which were detected in all human tissues, only 11% of lncRNAs were detected in these tissues, which suggested that lncRNAs show more tissue-specific expression patterns.^79^ The expression of lncRNA in T cell lineages is a good example of its cell- and developmental stage–specific expression patterns. Hu et al. conducted a pair-wise comparison of protein-coding genes and lncRNAs between different stages of T cell development. Their results indicated that mRNAs are similarly expressed between different T cell subsets, while remarkably different lncRNAs were expressed between various T cell subsets. Quantitative analysis showed that 48–57% of lncRNAs, in contrast to 6–8% of coding genes, were specifically expressed in various T cell subsets.^80^ This was further proved by other studies. By profiling lncRNA expression of CD8^+^ T cell subsets in both humans and mice, researchers found that lncRNA-Snhg1, which exhibits the naive^hi^-effector^lo^-memory^hi^ expression pattern, plays an essential role for memory CD8^+^ T cell establishment. Thus lncRNA-Snhg1 could be a unique biomarker to identify this subset of T cells.^81^ Certain ncRNAs are also candidate biomarkers for predicting therapy resistance.

Considerable attention has been paid to the use of non-invasive methods such as liquid biopsies to analyze biomarkers from body fluids (e.g., blood, saliva, urine). The reliability and reproducibility of these assays to detect and characterize tumors have tremendous value with far-reaching clinical implications. The use of biomarkers in body fluids to predict cancer therapy response has made significant progress, allowing for the selection of appropriate treatment options.^82^ Biomarkers that are easily accessible from body fluids are circulating tumor cells, circulating proteins, DNA, and RNA, including ncRNAs. Circulating RNAs are largely secreted by cells and therefore give hints regarding diseases and biological processes, including response to therapy. Harnessing the role of certain ncRNAs in intrinsic and acquired treatment resistance has led to their study as biomarkers that can predict therapeutic outcomes in a given patient before, during, or after treatment. This association is partially dependent on the property of ncRNAs to function in cell-to-cell communication, mediating drug resistance.^83^

NcRNAs can travel in body fluids in three different forms: bound to proteins, bound to lipoproteins, or inside small EVs. The mechanisms are especially well described for miRNAs. NcRNAs form RNA-protein complexes, can be released by cells, and are probably the predominant mechanism of cell-to-cell communication. Argonaute complexes, the pivotal component of the miRNA-induced silencing complex formed inside cells, contribute to the stability of plasma miRNAs by binding them.^84^ Lipoproteins such as high- and low-density lipoproteins (HDL and LDL) are inherently soluble and have the tendency to embed water-insoluble material inside their core, which enables them to transport nucleic acids between cells and also protects miRNAs from degradation by RNases.^85,86^ These miRNAs are then transferred to recipient cells and can regulate downstream gene expression. Another interesting form of cell-to-cell communication is mediated by EVs. Exosomes, the smallest subclass of EVs, have been extensively investigated recently in cancer pathogenesis.^87^ They are produced via exocytosis of multivesicular bodies that enclose various types of molecules, including ncRNAs, and are secreted into the interstitial spaces circulating in body fluids. By endocytosis of ncRNAs enclosed into exosomes of neighboring or remote recipient cells, cell signals can be transferred between cells, including the drug-resistant phenotype.^88^

The role of ncRNAs as critical regulators of carcinogenesis and therapeutic resistance is supported by in vivo and in vitro data, and the focus of this section is to discuss ncRNAs as biomarkers to predict response in cancer therapy.

### Chemotherapy resistance

Multidisciplinary cancer treatment is being effectively used worldwide. Though chemotherapy is one of the traditional standard approaches for cancer management, only a fraction of patients will experience objective clinical response to various chemotherapy regimens. Therefore, characterizing novel biomarkers to discriminate patients who are intrinsically resistant to the planned chemotherapy will avoid unnecessary adverse side effects. 5-FU and oxaliplatin are two fundamental chemotherapy agents that are components of the most common chemotherapy regimens for CRC and other gastrointestinal cancers, e.g., FOLFOX, FOLFIRI, and XELOX. By screening the differentially expressed miRNAs from 20 matched CRC serum samples with or without objective response to oxaliplatin-based chemotherapy, Zhang et al. identified five miRNAs—miR-20a, miR-130, miR-145, miR-216, and miR-372—that were significantly downregulated in responders compared to non-responders. The area under the receiver operating characteristic curve (AUC) values of this group of miRNAs in the training and validation set comprising of 40 and 173 samples were 0.841 (95% CI: 0.707–0.975) and 0.918 (95% CI: 0.871–0.963), respectively. This miRNA signature also demonstrated better accuracy in predicting chemotherapy resistance than traditional tumor biomarkers such as CEA (AUC = 0.689, 95% CI: 0.618–0.0.760), and CA19-9 (AUC = 0.746, 95% CI: 0.682–0.851).^89^ However, whether the serum samples were obtained before or after the initiation of the treatment is unclear.

Similarly, patients with metastatic CRC who were resistant to first-line 5-FU/oxaliplatin-based chemotherapy showed higher expression of miR-130b, miR-106a, and miR-484 compared to responders. The data were further validated in another cohort of 150 patients.^90^ Of note, the plasma samples were obtained prior to treatment, suggesting that these plasma miRNAs may serve as non-invasive markers to predict intrinsic resistance to 5-FU and oxaliplatin–based chemotherapy in metastatic CRC patients. In another study, of 742 miRNAs profiled in metastatic CRC patients who did and did not respond to XELOX/FOLFOX, high expression of miR-625-3p was correlated with poor response; this finding was validated in a cohort of 94 patients (OR = 6.25, 95% CI:1.8–21.0). However, miR-625-3p was not associated with prognosis, suggesting that miR-625-3p might solely be a response-predicting biomarker. miR-625-3p was also overexpressed in an oxaliplatin resistance–induced HCT116 cell line compared to parental cells.^91^ MAP2K6-p38 signaling might be involved in the induction of this resistance.^92^

MiR-20a, miR-145, and miR-106a are also widely acknowledged as key miRNAs in chemotherapy resistance.^93,94^ miR-20a-5p regulates chemosensitivity to gemcitabine by targeting ribonucleotide reductase subunit M2 in pancreatic cancer and predicts the response to gemcitabine-based chemotherapy with satisfying predictive value (AUC = 0.89).^95^ Upregulation of miR-20a and downregulation of miR-451 after the second cycle of neoadjuvant chemotherapy, which is widely applied to treat locally advanced breast cancer, predicted resistance to treatment in HR^+^/HER2^-^ breast cancer (AUC = 0.80 and 0.788, respectively).^96^ Though miR-20a was associated with chemoresistance and radioresistance in in vitro and in vivo studies, these findings were not validated in independent patient cohorts.^97–99^ In a small cohort of triple-negative breast cancer patients (*n* = 32) who received neoadjuvant cisplatin/doxorubicin-based chemotherapy, miR-145-5p was downregulated in patients who achieved pathological complete response. The AUC of miR-145-5p as the predictor for response in this cohort was 0.7899 (95% CI: 0.6382–0.9416). It is plausible that miR-145 inhibited cell proliferation by targeting TGFβR2.^100^ In another study including 57 luminal breast cancer patients who received neoadjuvant chemotherapy, the level of miR-145 was significantly lower in responders compared to non-responders.^101^ Lim et al. performed miRNA sequencing in 1362 childhood acute myeloid leukemia samples, which comprised 1303 primary, 22 refractory, and 37 relapse samples. By applying differential expression analysis, they found that miR-106a-3p and miR-106a-5p could be biomarkers of treatment resistance, as these two miRNAs were consistently overexpressed in treatment-resistant samples—that is, refractory or relapse samples, and in primary samples from patients with induction failure. Further integrative miRNA:mRNA analysis found that miR-106a targeted the genes associated with oxidative phosphorylation, which is suppressed in treatment-resistant conditions.^102^ In addition, miR-9-5p, miR-9-3p, miR-433-3p, miR-21, and miR-200c may possess potentially predictive roles in chemotherapy resistance in GC and esophageal cancer (EC).^103–105^

LncRNAs also participate in the development of chemoresistance and may serve as potential biomarkers in CRC. In a cohort comprising 140 CRC patients, the lncRNA *XIST* was upregulated in patients who showed no response to 5-FU compared to those who showed response. These findings were validated in serum samples from 120 CRC patients from the same cohort with an AUC, diagnostic sensitivity, and specificity of 0.756, 71.7%, and 68.3%, respectively. Mechanistically, in vivo studies revealed out that X*IST* restrained 5-FU–induced cytotoxicity by promoting thymidylate synthase, a pivotal target of 5-FU.^106^ Similarly, both tissue and serum *MEG3* were downregulated in oxaliplatin-resistant CRC patients. *MEG3* showed potential to screen out non-responders, with an AUC of 0.784, the diagnostic sensitivity of 72.86%, and specificity of 61.43%.^107^ In these studies, the expression levels of lncRNAs were investigated in tissues and corresponding serum samples, demonstrating the consistency of their prognostic ability and their potential as candidate biomarkers. In a comprehensive profiling study with training and testing datasets including 1102 patients, a three-lncRNA signature (*AK291479*, *U79293*, and *BC032585*) was identified to predict pathological complete response after neoadjuvant chemotherapy in breast cancer.^108^ Liu et al. assigned different weights to the expression levels of eight lncRNAs expressed by 258 high-grade serous ovarian cancer patients from The Cancer Genome Atlas (TCGA) and successfully generated a risk-score formula for predicting chemotherapeutic sensitivity.^109^

### Targeted therapy resistance

#### Angiogenesis inhibitors

MiR-126, specifically expressed in endothelial cells, plays a pivotal role in the regulation of blood vessel integrity, which might affect anti-angiogenic treatment.^110^ Hansen et al. reported that plasma miR-126 was dynamically increased during the treatment of patients whose metastatic CRC was resistant to first-line XELOX chemotherapy combined with bevacizumab, suggesting that miR-126 may serve as a predictive biomarker for acquired resistance to chemotherapy or bevacizumab during treatment.^111^ Whether chemotherapy or bevacizumab or both are regulated by miR-126 remains unknown, as anti-angiogenic therapy usually is prescribed with other combined modality therapies but not by itself. miR-126 was also reported to be involved in multi-drug resistance through a variety of mechanisms, e.g., contributing to sorafenib resistance.^112^ miR-126 is also well known for endowing leukemia stem cells with chemotherapy resistance ability. It was significantly upregulated in relapse blasts compared to paired diagnostic samples and also after induction or salvage chemotherapy in acute myeloid leukemia patients.^113^ In contrast, there was an inverse correlation between the level of miR-126 and acquired resistance to dabrafenib in melanoma and tamoxifen treatment in estrogen receptor-positive breast cancer, suggesting multiple roles for the same miRNA in different therapies.^114,115^ Rinnerthaler et al. divided two cohorts of breast cancer patients treated with chemotherapy with or without bevacizumab into responder and non-responder groups according to the length of progression-free survival and then selected the differentially expressed miRNAs between the two groups. By identifying the mutually differentially expressed miRNAs and the miRNAs with prognostic power from these two cohorts they selected 12 miRNAs that provide survival information. Finally, in a validation cohort of 230 patients from a randomized trial, they confirmed that low expression of miR-20a-5p was the only predictor of benefit from bevacizumab-containing therapy.^116^ Interestingly, decreased expression of the same miRNA, miR-20a, in CRC positively correlated with treatment response with oxaliplatin-based chemotherapy, indicating miRNA specificity for treatment and disease state,^89^ which could be attributed to molecular mechanisms that govern the disease and site of action. However, predictive measures such as the AUC, sensitivity, and specificity of using these miRNAs need to be further investigated.

#### Tyrosine kinase inhibitors

The unique histological and molecular features of lung cancer, especially NSCLC, have offered considerable promise for precise personalized medicine in multidisciplinary cancer management. This has been made possible because of tremendous efforts that unraveled the underlying molecular mechanisms, particularly the discovery of mutations and/or alteration of genes such as *EGFR*, *ALK*, and *ROS1*. Despite EGFR TKIs’ selectively targeting EGFR-mutant NSCLC with significant treatment response, 20–30% of patients either do not respond or respond for less than 3 months; these are considered to have intrinsic resistance to treatment.^43^ By profiling the different miRNAs in gefitinib-sensitive and -resistant samples with EGFR mutation, miR-25, miR-122, miR-195, miR-21, and miR-125b were identified to predict gefitinib sensitivity in EGFR-mutated NSCLC.^117^ The AUC (0.869) of the combination of these plasma miRNAs had shown a discriminatory power of detecting EGFR mutation. This could be an indication for using plasma EGFR analyses of cell-free DNA when it is infeasible to get tissue samples to detect EGFR mutation status. However, no validation of the predictive value of this panel miRNAs in predicting the intrinsic resistance for EGFR-TKI was performed. Furthermore, except for the primary and secondary T790M mutation, mechanisms contributing to the resistance of EGFR-TKI have not been fully explored. Besides its role in inducing oxaliplatin resistance in CRC, miR-625-3p was also reported to induce a T790M-indepedent acquired resistance by activating the TGF-β/Smad pathway and EMT in vitro.^118^

Secondary imatinib resistance is the major reason for therapeutic failure in GISTs and poses a huge clinical challenge. The level of serum miR-518e-5p is higher in patients with GIST and secondary imatinib resistance than those with imatinib-sensitive GIST. The AUC, sensitivity, and specificity of miR-518e-5p to predict response to imatinib were 0.9938, 99.8%, and 82.1%, respectively, which demonstrated a satisfactory ability to discriminate the resistant tumors.^119^ Around 65% of patients have intrinsic resistance to bortezomib and do not respond to treatment with this widely used targeted therapy for multiple myeloma.^120^ The integrated expression of miR-215-5p, miR-181a-5p, and miR-376c-3p, with an AUC of 0.95 (95% CI: 0.84–1.00), could discriminate between patients with refractory versus sensitive multiple myeloma treated with bortezomib.^121^ The miRNA signature model identified in this study could serve to enhance the rate of treatment success.

### Radiotherapy resistance

EC patients who cannot undergo esophagectomy receive concurrent chemoradiotherapy as the alternative standard treatment, but only 30–50% achieve a permanent response. Radioresistance has been implicated in the upregulation of miR-193b, which increases the proportion of cells in the G0/G1 phase. Serum miR-193b was significantly lower in patients who had a complete response than in those who exhibited a partial response after radiotherapy, and it had a good predictive value for detecting EC patients who achieved a complete response (AUC = 0.710, 95% CI: 0.580–0.839).^122^ Besides the expression level itself, single nucleotide polymorphisms of ncRNAs or associated regulatory regions also correlated with radiosensitivity. For example, rs4938723 in the promoter region of miR-34b/c was related to chemoradiotherapy response in EC. Data from 175 patients showed that patients with the CC rs4938723 genotype had a better response to chemoradiotherapy than that of patients with TT or TC genotypes. The predictive model showed an AUC, sensitivity, and specificity of 0.777, 85.1%, and 71.3%, respectively, which was considered promising for EC patients.^123^

In patients with locally advanced rectal adenocarcinoma, an lncRNA signature comprising *lnc-KLF7-1*, *lnc-MAB21L2-1*, and *LINC00324* was validated to predict the response to neoadjuvant chemoradiotherapy, with good performance (AUC = 0.93).^124^

### Immunotherapy resistance

Identifying clinical biomarkers that can accurately predict the response to immunotherapy remains a significant challenge for the widespread application of ICI. Depending on the type of ICIs, immunohistochemistry expression of PD-L1/PD-1 on tumor cells and immune cells and tumor mutation burden (TMB) have emerged as promising biomarkers for predicting response to immunotherapy. Expression of PD-L1 by immunohistochemistry in tumor samples was approved by the US Food and Drug Administration (FDA) to be the criteria for the use of some ICIs, e.g., the indication for pembrolizumab in treating metastatic NSCLC. However, the requirement of biopsies and imprecise assessment of the results due to the intratumor heterogeneity limits its application. Thus, ncRNAs, especially the circulating ones that directly and indirectly target immune checkpoint molecules such as PD-1/PD-L1, TIM3, CTLA-4, B7-H3, and LAG-3, can be also implicated as biomarkers with great potential.^125–127^ There is a correlation between high TMB and response to ICIs in microsatellite instability high metastatic CRC.^128^ High TMB represents a high abundance of neo-epitopes that arise from the modification of proteins encoded by mutated genes, which leads to the activation of anti-cancer immune responses against those neoantigens. The survival of patients with head and neck squamous cell carcinoma has recently been prolonged with the implementation of ICIs. Therefore, Xia et al. explored whether a 25-miRNA-based classifier from the head and neck squamous cell carcinoma cohort in the TCGA database can predict TMB levels to identify patients who truly benefit from ICIs. The AUCs of this 25-miRNA-based signature model to predict TMB status were 0.822 for the training set, 0.702 for the test set, and 0.774 for the total set.^129^ Similarly, in uterine corpus endometrial carcinoma, the AUCs of a 26-miRNA signature for predicting TMB were 0.869 for the training set, 0.904 for validation the set, and 0.820 for the total set. This miRNA signature pattern also correlated with the expression of PD-1 and PD-L1, mismatch repair-related genes such as *MLH1* and *MSH6*, and homologous recombination repair of double-strand DNA break genes such as *BRCA1* and *BRCA2*.^130^ A similar study was reported in lung adenocarcinoma.^131^ By analyzing the TCGA data for colon cancer, a multi-lncRNA signature including 14 lncRNAs for predicting TMB levels was established. This combined-classifier had better efficiency to predict TMB—with AUC levels at 0.70, 0.71, and 0.71 in three validation sets—than the traditional clinical characteristics.^132^ Another 33-lncRNA–based signature classifier was developed in stomach adenocarcinoma to predict TMB, with outstanding performance.^133^

In addition to identifying TMB, miRNAs are used as indirect biomarkers of response to ICI therapy. A phase 2 study that explored the efficacy of nivolumab, a PD-1 inhibitor, in esophageal squamous cell carcinoma revealed that serum miR-1233-5p levels (AUC = 0.895) before nivolumab treatment and miR-6885-5p, miR-4698, and miR-128-2-5p levels (AUC of 0.93, 0.97, and 0.93, respectively) after treatment initiation predicted response to ICI.^134^ Though this was a small study, the evidence indicates the usability of ncRNAs for future prospective clinical trials. By investigating the differences of pretreatment circulating miRNAs between responders and non-responders in patients with NSCLC who received anti–PD-1 immunotherapy, Shukuya et al. developed a response-predicting miRNA signature that consists of miR-199a-3p, miR-21-5p, and miR-28-5p. This combination had better efficiency to predict anti–PD-1 immunotherapy response—with an AUC of 0.925, which is superior to the PD-L1 expression score determined by immunohistochemistry (AUC = 0.575).^135^

The tumor microenvironment is populated by multiple types of immune cells: T cells, macrophages, MDSCs, and NK cells that regulate the response to immune therapy. The ncRNAs that affect the function of these essential immune cells can be implicated in predicting the response to immune therapy.^136^ By targeting the transcription factor T cell factor 1 (TCF1), the key regulator of effector T cells, miR-24 modulates the immune response by controlling cytokine production of T cells. Besides these, some miRNAs can be exchanged via exosomes between T cells and antigen-presenting cells during antigen recognition to mediate the immune interactions and orchestrate the immune response. It is reasonable to propose that these miRNAs can be alternative candidates to predict immunotherapy response.^137^

A selected list of ncRNAs with potential value in monitoring resistance to chemotherapy, radiotherapy, targeted therapy, and immunotherapy is presented in Table 2.

**Table 2:** miRNA and lncRNA with potential roles in predicting therapy response and diagnosing resistance to cancer treatment

Cancer type	ncRNAs	Function in therapy resistance	Sample type	Screening/training cohort sample size	Validation cohort sample size	SE/SP (training cohort; validation cohort)	AUC (training cohort/validation cohort)	Ref
*Resistance to chemotherapy*								
CRC	A signature including miR-20a, miR-130, miR-145, miR-216, and miR-372	Downregulated in responders to oxaliplatin-based regimen	Serum	40/40	173	NA	0.841/0.918	^89^
mCRC	miR-130b, miR-106a, miR-484	Overexpressed in patients with resistance to first-line 5-FU/oxaliplatin-based chemotherapy	Plasma	24	150	NA	NA	^90^
mCRC	miR-625-3p	Overexpressed in patients with poor response to XELOX/FOLFOX	Tumor tissue	26	93	NA	NA	^91^
Pancreatic cancer	miR-20a-5p	Abundant level predicts gemcitabine resistance	Plasma	73	NA	NA	0.89	^95^
HR^+^/HER2^‐^ breast cancer	miR-222, miR-20a, miR-451	Overexpression of baseline miR-222, and upregulation of miR‐20a, and downregulation of miR-451 after 2^nd^ cycle predict resistance to neoadjuvant chemotherapy	Plasma	6	51	NA	0.706 for miR-222; 0.800 for miR-20a; 0.788 for miR-451	^96^
Triple-negative breast cancer	miR-145-5p	Downregulated in patients who achieved pCR to cisplatin/doxorubicin-based chemotherapy	Tumor tissue	32	NA	NA	0.7899	^100^
Luminal breast cancers	miR-145	Decreased in patients who respond to neoadjuvant chemotherapy	Serum	56	NA	NA	NA	^101^
Pediatric AML	miR-106a-3p/5p	Overexpressed in induction chemotherapy-resistant patients	NA	637	666	NA	NA	^102^
GC	A signature including miR- 9-5p, miR-9-3p, and miR-433-3p	Overexpressed in cisplatin-resistant patients	Serum	74	NA	0.80/0.79	0.915/NA	^103^
Metastatic GC	miR-21	Overexpressed in chemotherapy-resistant patients	Tumor tissue, plasma	92	NA	0.88/0.69	0.83	^104^
EC	miR-200c	Overexpressed in chemotherapy-resistant patients	Tumor tissue	98	NA	NA	NA	^105^
CRC	LncRNA XIST	Upregulated in patients with poor response to 5-FU	Tumor tissue, serum	10/140	120	0.72/0.68	NA/0.756	^106^
CRC	MEG3	Downregulated in CRC patients showing no response to oxaliplatin	Tumor tissue, serum	8/160	140	0.72/0.61	NA/0.784	^107^
Breast cancer	A signature including AK291479, U79293, and BC032585	Upregulation of lncRNAs AK291479 and BC032585 and downregulation of U79293 in patients with pCR to chemotherapy	Tissue	488	614	NA	0.74 /0.72	^108^
Ovarian cancer	A signature including eight lncRNAs	An eight-lncRNA signature associated with chemosensitivity to cisplatin	Tissue	NA/258	233	NA	0.83/0.67	^109^
*Resistance to target therapy*								
Angiogenesis inhibitors								
mCRC	miR-126	Upregulated in patients with resistance to XELOX chemotherapy combined with bevacizumab	Blood	63	NA	NA	NA	^111^
ER-positive breast cancer		Increased in patients with response to tamoxifen treatment	Tumor tissue	12	81	NA	NA	^115^
Metastatic breast cancer	miR-20a-5p	Lower expression predicts benefit from bevacizumab	Tumor tissue	115	203	NA	NA	^116^
TKI and other small molecular inhibitors								
Lung cancers	A signature including miR-195, miR-122, miR-125, miR-21, and miR-25	This signature can predict the EGFR mutational status and gefitinib sensitivity	Tumor tissue, plasma	35	149	NA	NA/0.869	^117^
GISTs	miR-518e-5p	Increased in patients with secondary resistance to imatinib	Serum	6	76	0.99/0.82	NA/0.9938	^119^
MM	miR-215-5p, miR-181a-5p, miR-376c-3p	Dysregulated in bortezomib-refractory patients	Serum	30	NA	0.95/0.91	0.95	^121^
*Resistance to radiotherapy*								
EC	miR-193b	Higher in patients who exhibited PR to radiotherapy	Serum	75	NA	NA	0.71	^122^
ESCC	rs4938723 in the promoter region of miR-34b/c	CC genotype favors a better response to CRT compared to TT + TC genotypes	Blood	175	NA	0.85/0.71	0.777	^123^
Locally advanced rectal adenocarcinoma	Signature including lnc-KLF7-1, lnc-MAB21L2-1, and LINC00324	Classifying pCR to neoadjuvant CRT	Tumor tissue	49	NA	0.91/0.94	0.93	^124^
*Resistance to immunotherapy*								
HNSCC	A 25-miRNA-based signature	Predict TMB levels	Tumor tissue (TCGA)	301	200	0.421/0.937; 0.36/0.863	0.822/0.702	^129^
UCEC	A 26-miRNA-based signature			311	207	0.682/0.915; 0.568/0.882	0.904/0.820	^130^
Lung adenocarcinoma	A 25-miRNA-based signature			267	177	0.77/0.96; 0.67/0.96	0.895/0.826	^131^
Colon cancer	A 14-lncRNA signature			195	195	NA	0.70/0.71	^132^
Stomach adenocarcinoma	A 33-lncRNA-based signature			261	87	0.83/0.96; 0.56/0.91	0.999/0.974	^133^
ESCC	miR-1233-5p before treatment and miR-6885-5p, miR-4698, and miR-128-2-5p after treatment	Lower levels of miR-1233-5p before treatment and of miR-6885-5p, miR-4698, and miR-128-2-5p after treatment predict better response to nivolumab	Serum	19	NA	NA	0.895; 0.93, 0.97;0.93,	^134^
NSCLC	Signature including miR-199a-3p, miR-21-5p, and miR-28-5p	Decreased in responders to anti-PD-1 or PD-L1 antibody	Plasma	29	21	NA	NA/0.925	^135^

*5-FU* Fluorouracil; *AML* Acute myeloid leukemia; *AUC* Area under the receiver operating characteristic curve; *CRT* Chemoradiotherapy; *EC* Esophageal cancer; *ER* Estrogen receptor; *ESCC* Esophageal squamous cell carcinoma; *FOLFOX* Folinic acid, fluorouracil and oxaliplatin; *GC* Gastric cancer; *GISTs* Gastrointestinal stromal tumors; *HER2* Human epidermal growth factor receptor 2; *HNSCC* Head and neck squamous carcinoma; *HR* Hormone receptor; *mCRC* metastatic colorectal cancer; *MM* Multiple myeloma; *NSCLC* Non-small cell lung cancer; *pCR* Pathological complete response; *PR* Partial response; *SE* Sensibility; *SP* Specificity; *TMB* Tumor mutation burden; *UCEC* Uterine corpus endometrial carcinoma; *XELOX* Capecitabine + oxaliplatin.

## Therapeutic strategies to target ncRNAs to overcome therapy resistance

**Fig. 3:**
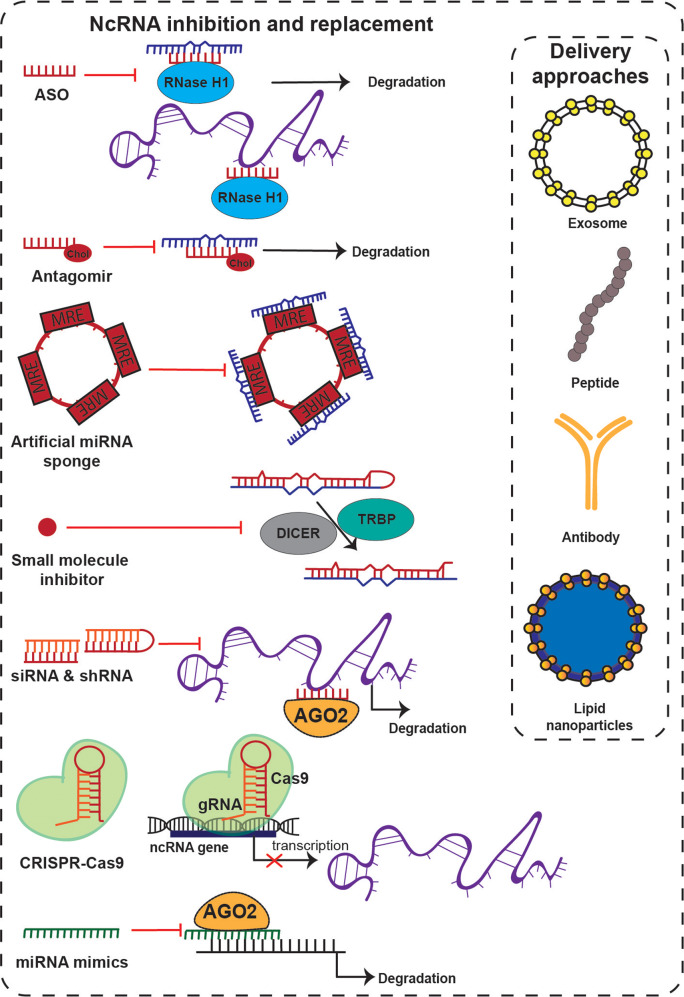
Therapeutic modalities to target ncRNAs. The therapeutic strategy to target overexpressed ncRNAs is to inhibit the specific ncRNA molecules. The inhibition modalities include (1) antisense oligonucleotides (ASOs): ASOs bind to complementary RNA sequences to block and inhibit their function and induce their degradation via RNAse-H-mediated cleavage; (2) antagomirs: antagomirs bind to complementary miRNAs and induce their degradation, thus preventing their interaction with target mRNA; (3) artificial miRNA sponges: artificial RNAs contain multiple high-affinity miRNA antisense binding sites that can sequester miRNAs from their target mRNAs; (4) small molecules: these molecules can interrupt any step of RNA transcription process; (5) small interfering RNAs (siRNAs) and short hairpin RNAs (shRNAs): these artificially synthesized double-stranded RNAs bind to complementary target ncRNA when loaded to AGO2, leading to the degradation of target RNA; (6) CRISPR/Cas9-based editing approaches, delivering the Cas9 nuclease complexed with a synthetic guide RNA (gRNA) to precisely cut the target ncRNA; and (7) miRNA mimics: miRNA mimics are used for replacing or substituting downregulated tumor suppressor miRNAs. Commonly used delivery systems of these ncRNA therapeutic modalities include lipid nanoparticles, exosomes, antibodies, and peptides.

Various RNA-based therapies have been developed, and some have been approved by the FDA. Of note, all these therapeutics target specific mRNAs to downregulate the expression of corresponding genes. Though lncRNAs have been the focus of recent investigations, none have been clinically investigated as therapeutic targets. The utility of miRNA-based therapeutics has been developed in phase 2 and 3 clinical trials. Therapeutic modalities targeting ncRNAs are usually developed with one of two strategies: the first is to inhibit the specific ncRNA molecule if it is overexpressed, and the second is to overexpress a tumor suppressor ncRNA.^138^ A schematic overview of the ncRNA therapeutic strategies and delivery mechanisms is depicted in Fig. 3.

NcRNA inhibitors include antisense anti-oligonucleotides (ASOs), antagomirs, siRNAs, short hairpin RNAs (shRNAs), miRNA sponges (including circRNA sponges), CRISPR/Cas9-based genome editing, and small molecule inhibitors of ncRNAs. ASOs and antagomirs are widely used inhibitors targeting miRNAs for in vitro and in vivo studies. ASOs are single-stranded RNA molecules that bind to complementary RNA sequences with well-matched base pairing to block and inhibit their function and induce their degradation via RNAse-H-mediated cleavage.^139^ Additionally, in preclinical in vivo studies, ASOs show specific and efficient reduction in lncRNA levels. Antagomirs are anti-miRNA ASOs that, when conjugated to cholesterol, show an improved intracellular delivery ability. Antagomirs function by complementary binding to miRNAs, thus preventing their interaction with their target genes.^140^ Locked nucleic acid is another commonly used chemical modification in anti-miRNA ASOs. Miravirsen (SPC3649) is a modified locked nucleic acid that is complementary to miR-122 and was investigated in phase 2 clinical trials for treating chronic hepatitis C infections.^141^ RNA interference is another commonly used strategy to degrade and knock down ncRNAs.^142^ SiRNAs are artificially synthesized double-stranded RNA molecules around 20 nucleotides long that function as RNA interference by complementary binding to their targets, leading to the transient silencing of gene expression.^143^ ShRNAs overcome the short lifespan of synthetic siRNAs, the main drawback, and are therefore widely employed in genetic screens and used as the common RNA interference approach in gene therapy and, occasionally, in clinical settings.^144^ CRISPR/Cas9 is a novel genome editing method that has been used to inhibit ncRNAs in preclinical in vivo and in vitro studies with considerable success.^145^ Artificial miRNA sponges are constructs containing multiple high-affinity miRNA antisense binding sites that target one specific or multiple different miRNAs.^146^ While the efficiency of miRNA sponging has been proven in in vivo studies, their utility in the clinic is still lacking. Small molecules either directly inhibit ncRNAs or indirectly target specific genes or proteins that regulate ncRNAs expression or function, usually involved in their biogenesis and maturation. Luciferase/GFP could be a strategy to effectively screen small molecular inhibitors that bind to mature miRNAs and block their binding to the miRNA response element.^147,148^ This interaction will then lead to the activation of luciferase/GFP, and the affinity could be preliminarily determined by the relative intensity of luciferase activity. Because lncRNAs exert their regulatory effect by interacting with RNA-binding proteins, small molecule inhibitors interfere with lncRNA-protein interactions and block lncRNA function.

The second strategy for targeting ncRNAs is to restore the normal function of ncRNAs that are downregulated when therapy resistance occurs. Function can be restored by replacing or substituting the lost ncRNA using synthetic ncRNA-like molecules such as miRNA mimic agents, an effective alternative widely used in in vivo studies.^149^ Despite remarkable progress in the field of ncRNA-based therapeutics, many challenges still need to be addressed—especially the issue of side effects caused by off-target effects.^138^ The well known miR-34 mimic MRX34 caused significant adverse events in five patients—with one patient suffering cytokine release syndrome—which led to the suspension of a phase 1 clinical trial for cancer treatment.^15^ Toll-like receptor signaling activated by miRNAs might explain this side effect: Toll-like receptors are activated, leading to the activation of downstream signaling nuclear factor-kappa B and then triggering the transcription and release of pro-inflammatory cytokines including IL-6, -8, -12, and TNF-alfa.^38,150^ Other non–immune-related off-target effects, due to mismatched base pairing to mRNAs that are not targets of interest, also need to be addressed. Another obstacle in RNA-based therapeutics, especially when the drug is systemically administered, is unexpected on-target effects on normal tissue but not the tumor tissue. For instance, ASO AEG35156 targeting the X-linked inhibitor of apoptosis gene also induced pathological peripheral neuropathy due to the on-target effect in neural system cells instead of cancer cells.^151^ Thus, the specificity, delivery, and tolerability of therapeutics using ncRNAs need to be further improved.

A safe and effective tissue delivery system for RNA-based therapeutic drugs without severe side effects remains one of the major challenges that limit their translational application. As mentioned above, antagomirs are anti-miRNA ASOs that are conjugated to cholesterol with improved intracellular entry affinity to targets. Such chemical modifications in the backbone, nucleobase, ribose sugar, and/or 2ʹ-ribose substitutions could increase the stability and efficacy of therapeutic oligonucleotides. However, an additional delivery system to enhance the affinity of the oligonucleotides is still warranted.^152^ Lipid nanoparticles are the most commonly used delivery system, with high biocompatibility and low toxicity.^153^ Liposomes are spherical nanoparticle vesicles consisting of double phospholipid layers resembling the structure of cell membranes. Liposomes are widely used to encapsulate hydrophilic or lipophilic drugs that target specific tissues.^154^ By avoiding the nuclease degradation and renal clearance of coated drugs, liposomes increase the cellular uptake of delivered drugs. Liposomes composed of ionizable lipid, phosphatidylcholine, cholesterol, or PEG-lipid conjugates have been successfully used in clinical trials, including the miRNA mimic MRX34 and patisiran. The latter agent is designed for treating hereditary transthyretin-mediated amyloidosis.^15,155^

A stimulus-responsive nanoparticle delivery system that releases the target drug in a stimuli-responsive manner (triggered by enzymes, pH, glutathione, specific temperature, hypoxia, ROS, etc.) was developed recently in order to increase the specificity of delivering the drug to the target tissue.^156^ This system has great advantages in increasing the homogeneous distribution and accumulation of the drug within target tumor tissue and therefore decreasing off-target effects. Moreover, physicochemical modifications to further increase its biocompatibility and spatial controls have also been employed. Gold stimulus-responsive nanoparticles, which can be easily synthesized and have a flexible size, are an ideal carrier for oligonucleotides. miR-124-5p^157^ and miR-145^158^ mimetics were recently explored to be encapsulated with these nanoparticles and shown to effectively target cancer cells. The release of these miRNA mimics is initiated by the cleavage of cystamine and then triggered by the high concentration of glutathione in the cytosol. Besides miRNAs mimics, circFoxo3 has been explored to be encapsulated within gold nanoparticles and delivered to target and induce apoptosis in melanoma cells.^33^ Triantennary N-acetylgalactosamine (GalNAc) can highly selectively bind to asialoglycoprotein receptor 1, which is highly expressed in the liver, making GalNAc ideal as a liver-targeted delivery system. When conjugated together with the oligonucleotides or siRNAs, GalNAc facilitates the uptake of these RNA drugs into hepatocytes by endocytosis with high selectivity.^159^ For example, givosiran, a GalNAc-conjugated siRNA, targets and downregulates 5ʹ-aminolevulinate synthase 1 to treat acute hepatic porphyria. Its efficacy was demonstrated in a phase 3 clinical trial.^160^ In addition, antibodies, aptamers, or peptides can be conjugated with siRNAs or ASOs for targeted delivery. Oligonucleotides conjugated to antibodies using click chemistry facilitate the degradation of complex after entry into the target cells, followed by releasing the ASO.^161^

Exosomes—EVs ranging from ~40 to 160 nm in diameter—have an endosomal origin and contain proteins, DNA, and RNA molecules. Cancer cells secrete exosomes that are delivered to distant cells to transmit intercellular messages. This intercellular communication mediated by exosomes containing ncRNAs has been proven to regulate drug resistance in various cancers.^88^ Because exosomes have better biocompatibility and biodistribution than synthetic delivery approaches, manipulating exosomes might hold promise as a delivery strategy in the clinic. Exosomes are natural biological nanoparticles offering unparalleled biocompatibility, and this property was harnessed in patients with severe therapy-refractory graft-versus-host disease, wherein exosomes from MSCs were safely delivered without causing severe immune reactions.^162^ Thus, each of the delivery strategies mentioned above could be a potential candidate for distributing ncRNA-based drugs to overcome therapy resistance. However, it should be noted that the unique design of a specific delivery system should be comprehensively based on the target ncRNA, tumor type, and other clinicopathological factors, as these features are essential to achieve the desired efficiency without causing severe off-target effects or toxicity.

## Future perspectives

Several ncRNAs are being studied in clinical trials as potential biomarkers for response to cancer therapy. A curated list of such ncRNAs is provided in Table 3.

**Table 3:** Clinical trials exploring ncRNAs as biomarkers of treatment response

Study Type	Conditions	Treatment	ncRNA	Sample type	Trial status	Trail identifier
Observational	Breast cancer	Chemotherapy ± hormone therapy	Circulating miRNA	Blood	Active, not yet recruiting	NCT01722851
Observational	Breast cancer	Hormone therapy	miRNA	Tissue	NA	NCT02950207
Observational	Metastatic breast cancer	Bevacizumab	miRNA	Blood	Terminated	NCT01598285
Interventional	TNBC	Epirubicin-cyclophosphamide plus paclitaxel-carboplatin	Circulating miRNA	Serum	Not yet recruiting	NCT04771871
Observational	Esophageal cancer	Chemotherapy/radiotherapy	Circulating miRNA	Plasma	Active, not yet recruiting	NCT02812680
Observational	GC	Capecitabine + cisplatin or capecitabine + oxaliplatin+/− trastuzumab	miRNA	Tissue and blood	Recruiting	NCT03253107
Observational	Medullary thyroid cancer	Vandetanib	miRNA	Tissue, blood	NA	NCT02268734
Observational	Prostate cancer	Androgen deprivation therapy	Exosomal miRNAs	Blood	Active, not yet recruiting	NCT02366494
Interventional	Metastatic castration-resistant prostate cancer	Androgen receptor target agents/LHRH agonist	Circulating miRNA	Blood	Recruiting	NCT04188275
Interventional	Metastatic castration-resistant prostate cancer	Chemotherapy/novel hormonal agent	miRNA	Blood	Recruiting	NCT04662996
Interventional	NSCLC	Radiotherapy	miRNA	Plasm	NA	NCT03074175
Observational	Ovarian cancer	Chemotherapy	miRNA	Plasma, urine, tumor samples	Active, not yet recruiting	NCT02758652
Observational	Pancreatic cancer	NA	miRNA	Serum	Recruiting	NCT04406831
Observational	Pancreaticobiliary cancers	Surgery/chemotherapy	CircRNA	Plasm	Recruiting	NCT04584996
Observational	Rectal cancer	Neoadjuvant chemoradiotherapy	miRNA	Blood	Recruiting	NCT03962088
Interventional	EGFR-driven advanced solid tumors	Dacomitinib	LncRNA	NA	Not yet recruiting	NCT04946968

*EGFR* Epidermal growth factor receptor, *GC* Gastric cancer, *LHRH* Luteinizing hormone-releasing hormone, *NSCLC* Non-small cell lung cancer, *TNBC* Triple-negative breast cancer.

Most cancer drugs are indicated for a specific histological tissue type. Novel therapies targeting molecular aberrations in multiple cancers have been developed only recently. This concept is termed tumor-agnostic cancer treatment and represents the future of personalized cancer therapy.^163^ For example, the tumor-agnostic drug pembrolizumab is a monoclonal antibody against PD-1 that was initially developed for melanoma patients but has been recently approved for microsatellite instability–high and mismatch repair–deficient tumors, regardless of the cancer type.^164^ The TKI entrectinib was approved by the FDA 2019 to treat ROS1-positive NSCLC but also any solid cancer that has a neurotrophic tyrosine receptor kinase gene fusion.^165^ Of note, several ncRNAs discussed in this review are dysregulated in multiple tumor types and, therefore, may be ideal next-generation targets as tumor-agnostic ncRNA- and RNA-based therapeutics. Because of their heterogeneous expression, ncRNAs can be the ideal markers/targets for a personalized therapeutic approach that overrides our histological and mutational understanding of cancers and brings it into a non-coding transcriptional era. We envision that multiple tumor types, especially after developing a therapy-resistant phenotype, will show a dysregulated ncRNA expression pattern that will indicate to the clinician the necessity to change the treatment regimen. The ncRNAs are actively secreted in bodily fluids, and therefore, this approach can also be used as a potential new liquid biopsy strategy. In addition to dysregulated expression, functional switches that will be detected by analyzing intracellular localization of the molecules should also be perceived as a signal of ncRNA-induced resistance. Basic research is moving from a histological (tissue based) and bulk molecular approach to a single cell approach. We want to point out that this step will not be sufficient for understanding the functions of ncRNAs and a subcellular approach will be necessary. This will be achieved only by performing a single cell spatial non-coding-transcriptomics, which we believe is the next methodological breakthrough that we need. Of note, the location in which miRNAs (the best studied ncRNAs) perform their regulatory function is still a matter of debate.^6^ Hence, we still need to answer fundamental questions, and we will probably be surprised when we will discover much more mature miRNAs with nuclear localization than expected. Finally, the development of a single cell sequencing technique for ncRNAs will also answer questions regarding ncRNAs’ intratumoral heterogeneity. Currently, single cell transcriptomics techniques can retrieve only poly-adenylated RNAs.^166^ For example, by using such a method in patients with triple-negative breast cancer and intrinsic resistance to neoadjuvant chemotherapy versus treatment-sensitive patients, Shaath et al. observed that lncRNAs can be used to cluster the patients in these subgroups and that five transcripts of the *MALAT1* gene are specifically upregulated in resistant patients.^167^ This kind of data shows us how much potential there is in moving ncRNA research to the single cell level and ultimately to the subcellular level.

An additional strength of ncRNAs is that these molecules are secreted in virtually all biological fluids. This makes them potential biomarkers, but unfortunately only few ncRNAs have been confirmed for this function. The reason behind is probably, again, heterogeneity, which is also the strength of ncRNAs as potential future biomarkers for personalized medicine. So, this field of research needs to be moved from the diagnostic/screening setting to a sub-classification and response to therapy setting. This type of research needs very well annotated cohorts of patients, which most previous studies lacked. The methodology needs also to be improved, by checking the expression of ncRNAs with specific bio-fluid localization (i.e., bound to proteins, bound to lipids, or intravesicular), the specificity of ncRNA diagnosis can be increased. We envision that, in the near future, ncRNAs could achieve clinical use as biomarkers, most probably in combination with complementary methods—for example, in combination with circulating tumor cells, protein biomarkers, or even metabolites.^168^

In summary, because ncRNAs are key regulators and predictors of cancer therapy resistance, they could function as therapeutic adjuvants and as components of a tumor-agnostic therapeutic strategy to improve anti-cancer response within existing therapeutic modalities, including chemotherapy, radiotherapy, ICIs, and targeted therapy. However, some key challenges remain and limit the clinical application for ncRNA therapeutics—including issues associated with tolerability, toxicity, and off-target effects, which need to be further elucidated.
